# Nurse-Led Diabetes Self-Management Education Improves Clinical Parameters in Ethiopia

**DOI:** 10.3389/fpubh.2018.00302

**Published:** 2018-10-23

**Authors:** Fikadu Balcha Hailu, Per Hjortdahl, Anne Moen

**Affiliations:** ^1^Faculty of Medicine, Institute for Health and Society, University of Oslo, Oslo, Norway; ^2^School of Nursing and Midwifery, Jimma University, Jimma, Ethiopia

**Keywords:** DSME, nurse-led, clinical outcomes, long-term glycemic control, type 2 diabetes

## Abstract

**Background:** Unlike in developed countries, the clinical effectiveness of diabetes self-management education (DSME) is not well-studied in the African context. Thus, this study sought to determine effects of DSME on clinical outcomes among type 2 diabetic (T2DM) patients in Ethiopia.

**Methods:** Before-and-after controlled study design was employed, with random assignment of 116 T2DM adult patients to a nurse-led DSME group and 104 to a treatment-as-usual (comparison) group. A nurse-led DSME with six sessions supported with illustrative pictures handbooks and fliers was customized to local conditions and delivered by trained nurses over 9 months. Our primary outcome was a change in the proportion of people with target glycated hemoglobin (HbA1c ≤ 7%). We used chi-square test and mixed model analysis.

**Results:** Seventy-eight (67%) and 64 (62%) participants assigned to intervention and comparison, respectively completed the study, and included in the final analysis. Mean HbA1c was significantly reduced by 2.88% within the intervention group and by 2.57% within the comparison group. However, change in the proportion of participants with target HbA1c and end-line mean HbA1c difference between the groups were not significant. Adjusted end-line fasting blood sugar (FBS), systolic blood pressure (SBP), and diastolic blood pressure (DBP) were significantly lower in the intervention group, by 27 ± 9 mg/dL, 12 ± 3, and 8 ± 2 mmHg, respectively.

**Conclusion:** After 9 months of nurse-led DSME, HbA1c was significantly reduced within both groups but there was no significant difference in HbA1c between groups. The intervention also showed some clinically significant effects on blood pressure and FBS.

**Clinical Trial Registration:** ClinicalTrials.gov Identifier: NCT03185689, retrospectively registered on June 14, 2017 on ClinicalTrials.gov. https://clinicaltrials.gov/ct2/show/NCT03185689.

## Introduction

Diabetes is increasing alarmingly in Africa and developing countries like Ethiopia, probably catalyzed by socioeconomic, demographic, nutritional transitions and childhood malnutrition (1–4). Ethiopia, the leading country in Africa, with over 2.6 million adults living with diabetes (5, 6). To postpone diabetes-related complications and premature deaths, empowering diabetic patients by introducing cost-effective DSME to diabetic care has been recommended (7–9).

DSME has been defined as the “ongoing process of facilitating the knowledge, skill, and ability necessary for prediabetes and diabetes self-care” (10). DSME focuses on making specific behavioral changes and enabling patients to develop effective problem-solving skills and self-efficacy (8). The ultimate goal of DSME is improving clinical outcomes, health status, and quality of life (10–12). Though, since there is no single, best approach of DSME, standards of DSME have been suggested (10).

Studies in developed countries reveal that DMSE is effective in glycemic control, blood pressure control, and weight management (13–19). However, the very limited research in Africa has resulted in conflicting evidence regarding the effectiveness of DSME on long-term glycemic control and other clinical outcomes. An intensive and structured DSME education given by nurses and physicians for 6 months showed significant reduction in HbA1c among those who received an intervention (20). However, group-based DSME led by ley health promoters in underserved urban South Africa did not show significant mean difference in HbA1c between groups. This study, however, showed significant mean systolic and diastolic blood pressures difference between the intervention and the control groups (21).

Effectiveness and adherence to of DSME may be affected by sociodemographic factors like education, gender, age, employment, onset of diabetes, and food insecurity (22–27). Diabetes education provided by combining group-based and one-to-one delivery strategy was reported to be more effective than either group-based or one-to-one (7). Diabetes education provided over longer time are also effective (7, 19, 28). Moreover, teach back strategies and use pictures recommended to better accommodate low literate diabetes patients add merit and value to these groups (29).

The effectiveness of DSME delivered by highly trained providers, measured by glycemic control and other clinical parameters, has been established in developed countries (21). Even though Africa has more diabetes-related premature deaths than any other continent, DSME for low-health-literate diabetic African populations in accordance with the local cultural, social, and economic context has not been well-studied. The challenges expected in developing countries like Ethiopia relates to a) people are less literate and b) health-care providers have limited understanding of the concept and application of health literacy (30). In Africa, nurse-led DSME adapted to the local culture and patients' literacy level could play an important role in reducing diabetes-related complications and premature deaths (31). Although Ethiopia has the largest number of adults with diabetes of any African country, to the best of our knowledge, DSME interventions have yet to be tested or implemented in the Ethiopian context.

The limited findings regarding the effectiveness of DSME on clinical parameters in Africa and Ethiopia indicate a need for controlled studies contextualized to the local situations that can accommodate low-literate diabetic patients. We conducted the present study to test nurse-led DSME model empirically and adjusted the model to accommodate care of low-literate T2DM patients in Ethiopia. Taking glycemic control gaged by HbA1c as a primary outcome, we aimed to increase the proportion of T2DM patients in the intervention group with controlled blood glucose by absolute 15%. Our secondary outcomes included mean differences in HbA1c, fasting blood sugar (FBS), SBP, DBP, body mass index (BMI), and waist circumference between the intervention and comparison groups.

## Methods and subjects

### Study design and setting

A controlled before-and-after study was undertaken in Jimma University Medical Centre (JUMC), Ethiopia.

### Study period

The baseline survey was conducted from February 2016 to May 2016, the DSME intervention was conducted from November 2016 to July 2017, and the end-line survey was conducted from August 2017 to October 2017.

### Sampling

The sample size was calculated using Epi info_7.exe with the assumptions of increasing the proportion of people with target HbA1c (≤7%) in the intervention group by absolute 15% with a power of 80%, and one-sided test at 0.05 significance level. Because HbA1c had never been done routinely in the hospital, we used FBS as a reference measure of glycemic control. Moreover, as compared to FBS test, HbA1c less is less likely to be affected by acute blood sugar fluctuations and recommended to indicate glycemic control relatively over a longer time. A cross-sectional study in the same hospital showed that 18% of diabetes patients had controlled FBS (32). With the addition of 15% contingency, from the total 447 adult T2DM patients on active follow-up we sought to recruit a total of 240 participants, and 220 agreed to participate in the study.

### Participant recruitment and treatment assignment

**Figure 1:**
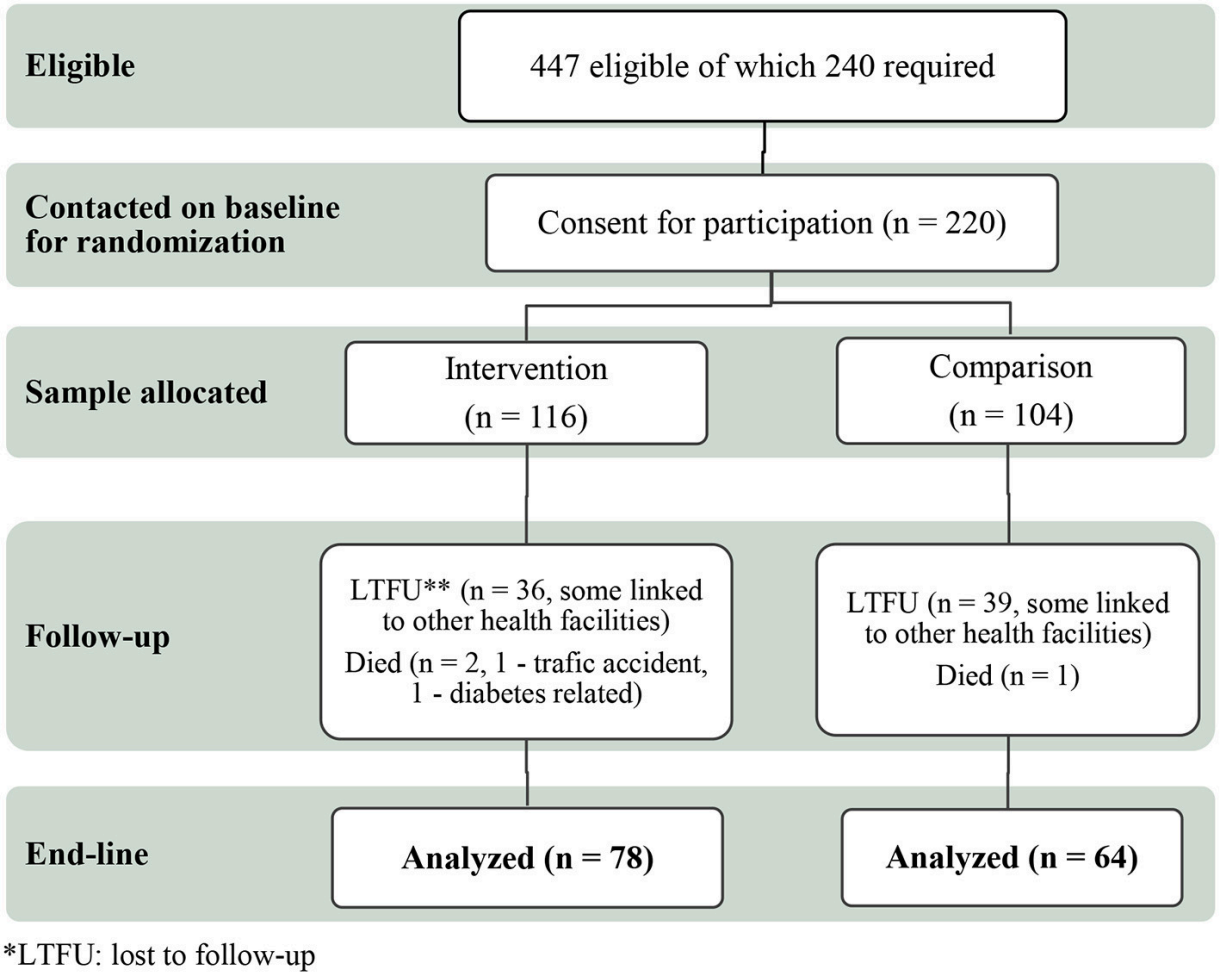
DSME consort flow chart.

Participants were recruited considering their residence, Jimma city and rural districts surrounding Jimma city in Southwest Ethiopia. Out of the total 220 participants included at baseline and given individual code number, we randomly assigned 116 to the DSME intervention and 104 to the comparison group, using the Excel random number generator. At the end-line, 78 from the intervention and 64 from the comparison participants were still in the study and included in the final analysis (Figure 1).

### Inclusion and exclusion criteria

We identified participants by the age at T2DM diagnoses (above 30 years), body weight (overweight or obese), or type of treatment (taking oral hypoglycemic agents [OHAs], record of intake of OHAs, or insulin and OHAs). Patients with type 1 diabetes or gestational diabetes, pregnant women, people with severe cognitive or physical impairment, and terminally ill people were excluded.

### Intervention

Participants assigned to the DSME treatment were grouped into 8–12 people per session. They participated in six DSME sessions for ~1.5 h every month for 6 consecutive months. For convenience, all the sessions were held on the date of the participants' routine follow-up, before they were seen by their doctors.

The DSME intervention was offered by PhD nurse student (FBH) and one clinical nurse fluent in the local languages Afan Oromo and Amharic, and they had been trained for a total of 16 h. The key role of the nurses was facilitating sessions.

**Figure 2:**
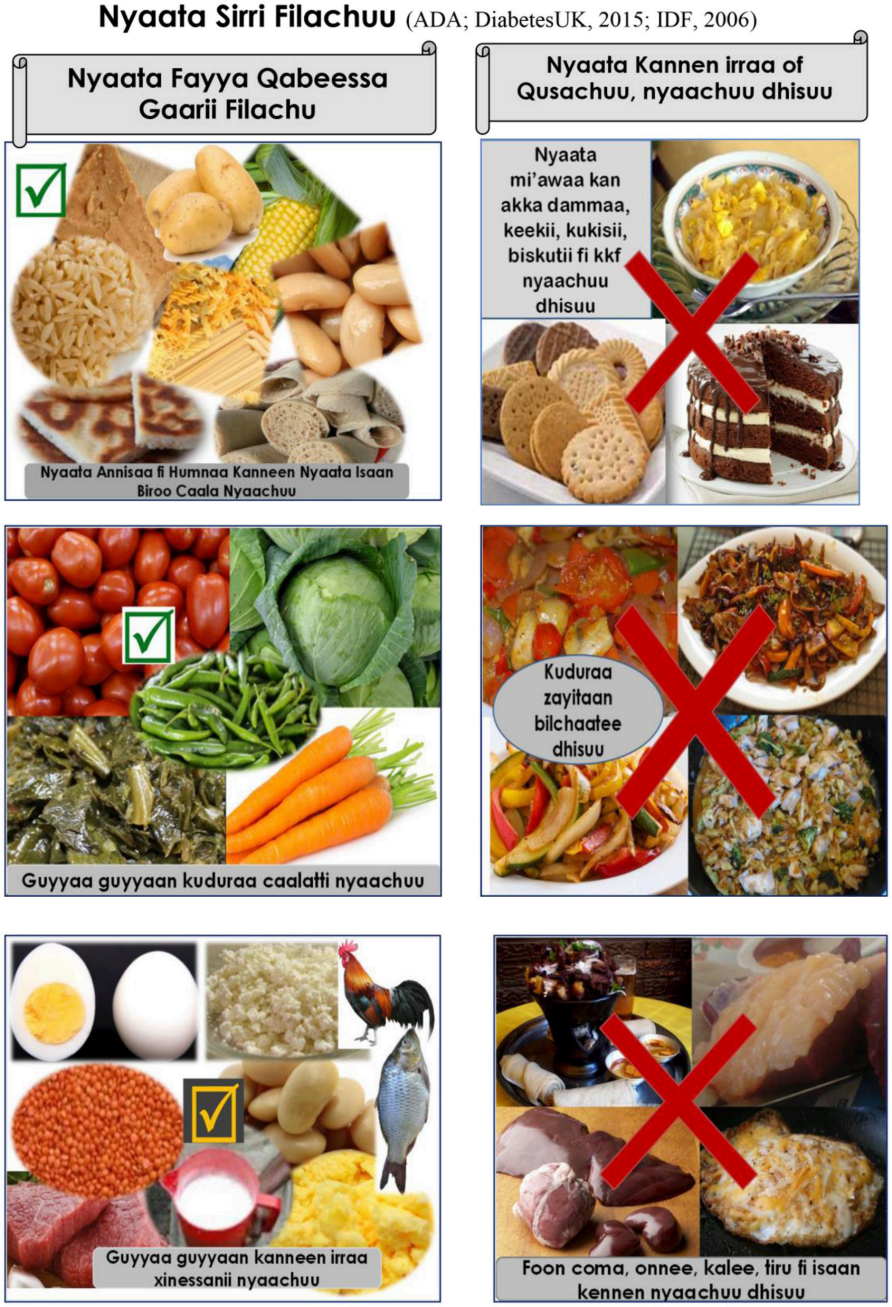
Excerpt from the Afan Oromo language instructional handbook: selecting a healthy diet.

**Figure 3:**
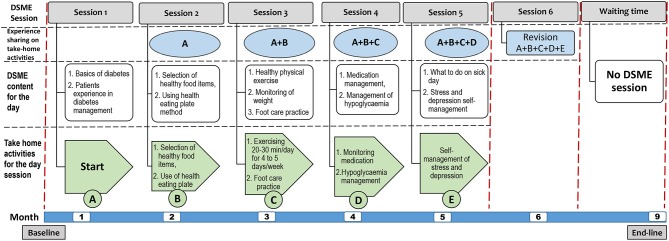
DSME sessions, daily topics, take-home activities, and experience-sharing areas.

The training was supported by handbooks and fliers with colorful, illustrative pictures customized to the local context and patients' literacy level (see Figure 2). Moreover, each of the 1.5 h DSME session had a series of interlinked activities including nurse facilitated brief education, discussion, experience sharing, take-home activities, conclusion and revision. During each DSME session, nurses facilitate brief education on the specific session topic, lead discussion, facilitate experience sharing among participants on the specific session topic, conclude session, give take-home activities from the specific session and on the next session start session of the day with revising how the participants were undertaking take-home activities (Figure 3).

We used the International Diabetes Federation training manual for Sub-Saharan Africa and other literature as point of departure to customize the DSME (12, 33–39). The content of the booklet was based on the seven key behaviors of DSME recommended by the American Associations of Diabetes Educators (40). For context-appropriate expertise, 27 T2DM patients (not included in the study) and other relevant stakeholders were consulted. The patient handbook was translated into two local languages: Afan Oromo and Amharic, and reviewed by bilingual experts from Jimma University, Ethiopia. The handbooks were distributed to participants during the first session, and fliers were distributed during each session.

The comparison group continued with usual follow-up care. After the end-line survey, the instructional handbook and fliers were also given to the comparison group. A random blood glucose (RBS) or FBS test was done for both groups during the follow-up period. To determine the effect of DSME sessions on glycemic control as gaged by HbA1c level, we measured HbA1c 3 months after the last DSME session.

To reduce loss to follow-up (LTFU), participants were reminded of upcoming sessions by phone, and on every visit, FBS test was provided free of charge for both groups. To reduce risk of information spillover, with the consent of patients we tried to vary the appointment dates of the groups, where the intervention group and the comparison groups would visit the hospital on different days.

### Data collection tool and technique

Data was collected by the trained nurses, using structured interviews, anthropometric measurements, and laboratory tests. One clinical laboratory technician performed lab tests. None of the data collectors were told about which group the participants were assigned to.

Height and weight were measured using a SECA stadiometer. Waist and hip circumference were measured using an inelastic tape measurement. Blood pressure was measured using an ACCOSON aneroid sphygmomanometer. For FBS measurement, a CareSens N I-sens glucometer was used, and for HbA1c, an ABX Pentra 400 HORIBA chemistry machine was used.

For household food insecurity, the household food insecurity access scale (HFIAS), validated in Ethiopia, was utilized (41). The questionnaire had nine occurrence questions, each of which was followed by a frequency-of-occurrence question (42).

### Data analysis

Data was fed in to EpiData entry client/manager (v. 4.2.0.0) and transported to StataSE 15 for analysis. Frequency distribution was run for proportion of participants with target HbA1c and FBS. A linear mixed model regression analysis was used to determine end-line mean differences in continuous outcome variables between groups. In the model, each of the outcome variables was adjusted for sociodemographic characteristics and patient-related clinical factors, including baseline targets of respective outcome variables. For the within group changes of the respective continuous outcome variables we used an independent sample *t*-test analysis.

Values of HbA1c ≤7%, FBS < 126 mg/dL, SBP < 140 mmHg, DBP < 90 mmHg, and waist circumference ≤94 cm for men and ≤80 cm for women were used as cut-offs (43–45). Households were considered food insecure if the response to occurrence question number 1 was “yes” and the response to the first (1a) frequency-of-occurrence question was “sometimes” or “often,” or if response to one of the occurrence questions 2–9 was “yes” (46).

For missing values of outcome variables, we used multiple imputation with the assumption of missing completely at random using twenty imputed data sets both at baseline and end-line. Intention-to-treat analysis was used to include participants who had at least an instructional handbook before the end of the intervention period and were assessed for the end-line. Accordingly, regardless of number of DSME sessions they attended, intervention group participants who had teaching hand book were contacted on the end-line survey were included in the final analysis.

## Result

### Sociodemographic characteristics

Out of the 240 T2DM patients planned to include in the study we were able to recruit 220 (116 participants in the intervention group and 104 in the comparison group) and they were included in the baseline analysis. At the end-line, 78 (67%) of the intervention and 64 (62%) of the comparison participants were included in the final analysis (Figure 1).

Out of 78 intervention group participants included in the final end-line analysis 6 (7.7%) attended no DSME session, 10 (12.8%) one, 7 (9.0%) two, 15 (19.2%) three, 10 (12.8%) four, 16 (20.5%) five, and 14 (18.0) six DSME sessions.

See Table 1 for the sample's baseline sociodemographic characteristics.

**Table 1:** Baseline sociodemographic characteristics of T2DM patients attending JUMC, May 2016.

**Variables**	**Intervention (*****n*** = **116)**		**Comparison (*****n*** = **104)**	
	**Frequency**	**Percent**	**Frequency**	**Percent**
Age (years) mean ± SD	55 ± 10		54 ± 10	
**GENDER**				
Male	81	70	67	64
Female	35	30	37	36
**EDUCATIONAL STATUS**				
Illiterate	20	17	23	22
Grade 1–6	33	28	27	26
Grade 7–12	39	34	33	32
College/university completed	24	21	21	20
**MARITAL STATUS**				
Married	98	84	85	82
Widow	10	9	10	9
Never married	6	5	3	3
Divorced	2	2	6	6
**MONTHLY FAMILY INCOME**				
Mean monthly family income in USD^*^ (SD)	93 ± 123		87 ± 77	
**FINANCIAL SOURCES FOR HEALTH CARE**				
Out of pocket	57	49	60	58
Paid by district	33	29	36	34
Insured	21	18	6	6
Other	5	4	2	2
**RESIDENCE**				
Urban	81	70	82	79
Rural	35	30	22	21
**FAMILY STRUCTURE**				
Nuclear family	89	77	76	73
Extended family	21	18	17	16
Living alone	6	5	9	9
With other individual	–	–	2	2
**HOUSEHOLD FOOD INSECURITY**				
Secure	52	45	53	51
Insecure	64	55	51	49

* *1USD at the time ~ 21.18 Ethiopian Birr*.

### Baseline clinical characteristics

At baseline, no significant variations were observed in clinical parameters between the intervention and comparison groups (Table 2).

**Table 2:** Baseline clinical characteristics of T2DM patients attending JUMC, May 2016.

**Variables**	**Intervention mean (SD)**	**Comparison mean (SD)**
Age at diagnosis (in years)	47 (10)	47 (10)
Years lived with diabetes	10 (6)	12 (7)
BMI (kg/m^2^)	25 (4)	25 (4)
Waist circumference (both sexes [cm])	93 (11)	96 (11)
Waist circumference (men [cm])	93 (10)	95 (10)
Waist circumference (women [cm])	94 (13)	98 (12)
Waist-to-hip ratio (both sexes)	0.96 (0.06)	0.96 (0.09)
Waist-to-hip ratio (men)	0.98 (0.06)	0.97 (0.08)
Waist-to-hip ratio (women)	0.94 (0.07)	0.94 (0.10)
SBP (mmHg)	124 (20)	125 (19)
DBP (mmHg)	79 (13)	78 (11)
FBS (mg/dL)	154 (61)	158 (65)
HbA1c (%)	11 (4)	10 (3)
**TYPE OF MEDICATION–FREQUENCY (%)**		
OHAs–frequency (%)	73 (63)	54 (52)
Insulin–frequency (%)	31 (27)	43 (41)
Both OHAs and insulin–frequency (%)	12 (10)	7 (7)

*BMI = weight (kg)/height (m)^2^*.

### Glycemic control

***HbA1c****: Chi-square* = *1.5795, P* = *0.208834*. ***FBS****: chi-square* = *1.554, P* = *0.212548* (baseline n: intervention = 116, comparison = 104; end-line n: intervention = 78, comparison = 64).

**Figure 4:**
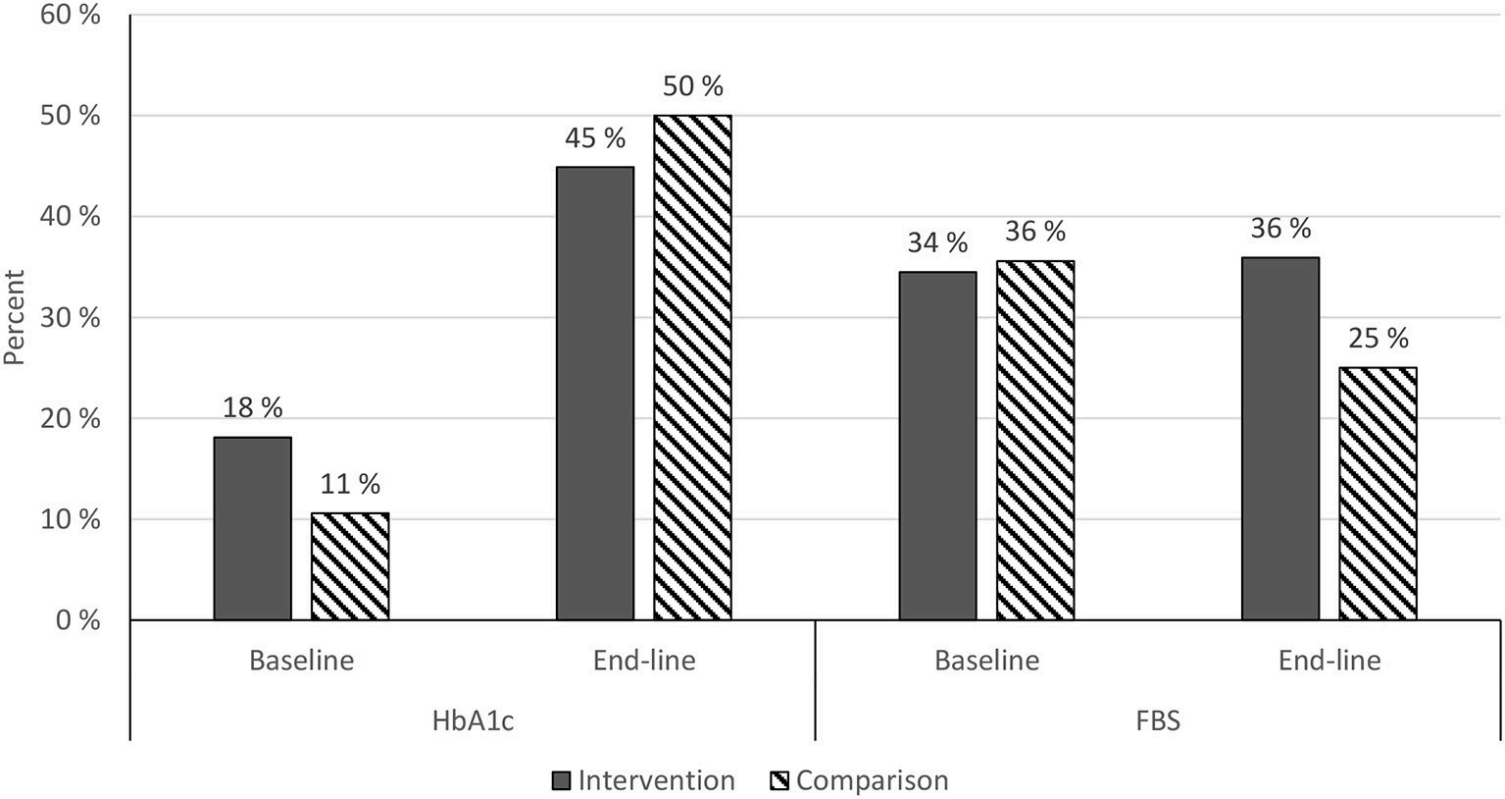
Target baseline and end-line HbA1c and FBS among JUMC diabetic patients, Oct. 2017.

Figure 4 shows an increased proportion of participants with end-line target HbA1c in both the intervention and comparison groups as compared to baseline HbA1c target (Figure 4).

**Figure 5:**
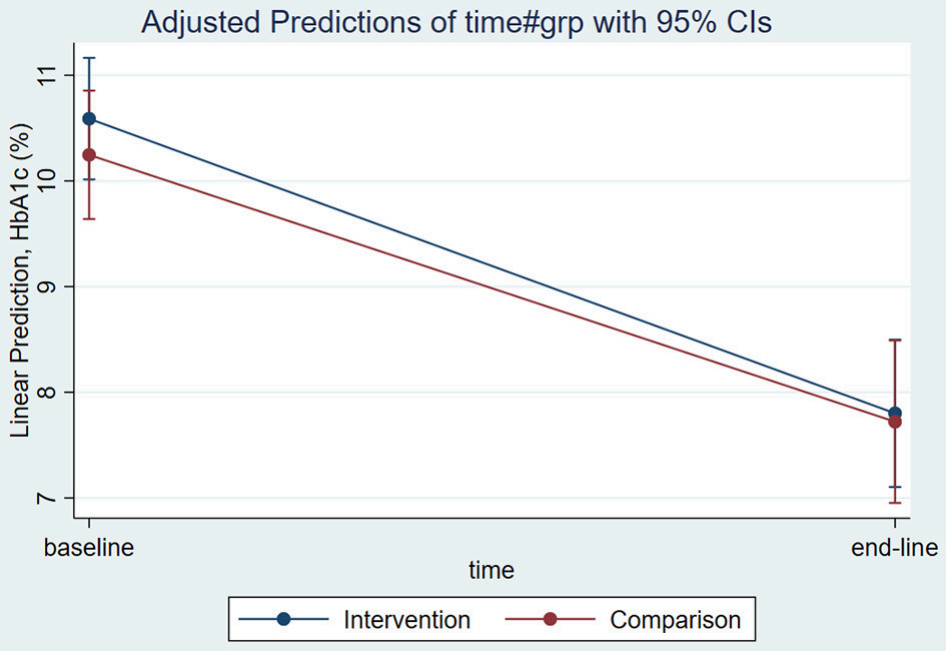
Adjusted mean end-line HbA1c among T2DM patients attending JUMC, Oct. 2017.

Using an independent sample *t*-test analysis HbA1c was reduced significantly by 2.88% (SD 4.28, CI: −3.85, −1.92) in the intervention group, and by 2.57% (SD 3.59, CI: −3.47, −1.67) in the comparison group from baseline to end-line., At end-line, the mean HbA1c difference between groups was not significant (Figure 5). Mean end-line FBS was significantly lower in the intervention group, by 27 ± 10 mg/dL (CI −45, −9; *P* = 0.003), when adjusted for sociodemographic and clinical factors, including baseline FBS target. However, there was no significant change in FBS within groups (Table 3).

**Table 3:** End-line fasting blood sugar mean difference between groups, Jimma Oct 2017.

**Fasting blood sugar**	**Coef**.	**Std. Err**.	**z**	***P* > *z***	**[95% Conf. Interval]**	
**TIME**						
Baseline	−14.70667	8.190383	−1.80	0.073	−30.75953	1.346182
**GROUP**						
Intervention	−27.28637	9.241271	−2.95	**0.003**	−45.39893	−9.17381
**TIME# GROUP**						
Baseline # intervention	20.15151	11.12643	1.81	0.070	−1.655893	41.95892
Age	0.0816722	0.3303327	0.25	0.805	−0.565768	0.7291123
Sex	13.10888	7.606254	1.72	0.085	−1.799102	28.01686
Educational status	2.227151	3.703041	0.60	0.548	−5.030677	9.484978
Marital status	−8.638841	4.975081	−1.74	0.082	−18.38982	1.112137
**RESIDENCY**						
Rural^*^						
Urban	−21.87865	8.591548	−2.55	**0.011**	−38.71778	−5.039527
Financial source	−6.404158	3.828788	−1.67	0.094	−13.90844	1.100128
Baseline household food insecurity	3.262884	6.118918	0.53	0.594	−8.729975	15.25574
Years lived with DM	−2.48475	4.094639	−0.61	0.544	−10.51009	5.540596
Type of medication	−6.751358	4.601998	−1.47	0.142	−15.77111	2.268392
**TARGET FBS AT BASELINE**						
Not target^*^						
Target	−68.1258	6.301119	−10.81	**0.000**	−80.47577	−55.77583
_cons	230.836	29.37252	7.86	0.000	173.2669	288.4051

* *Reference. Bold values: p < 0.05*.

### Blood pressure control

**Figure 6:**
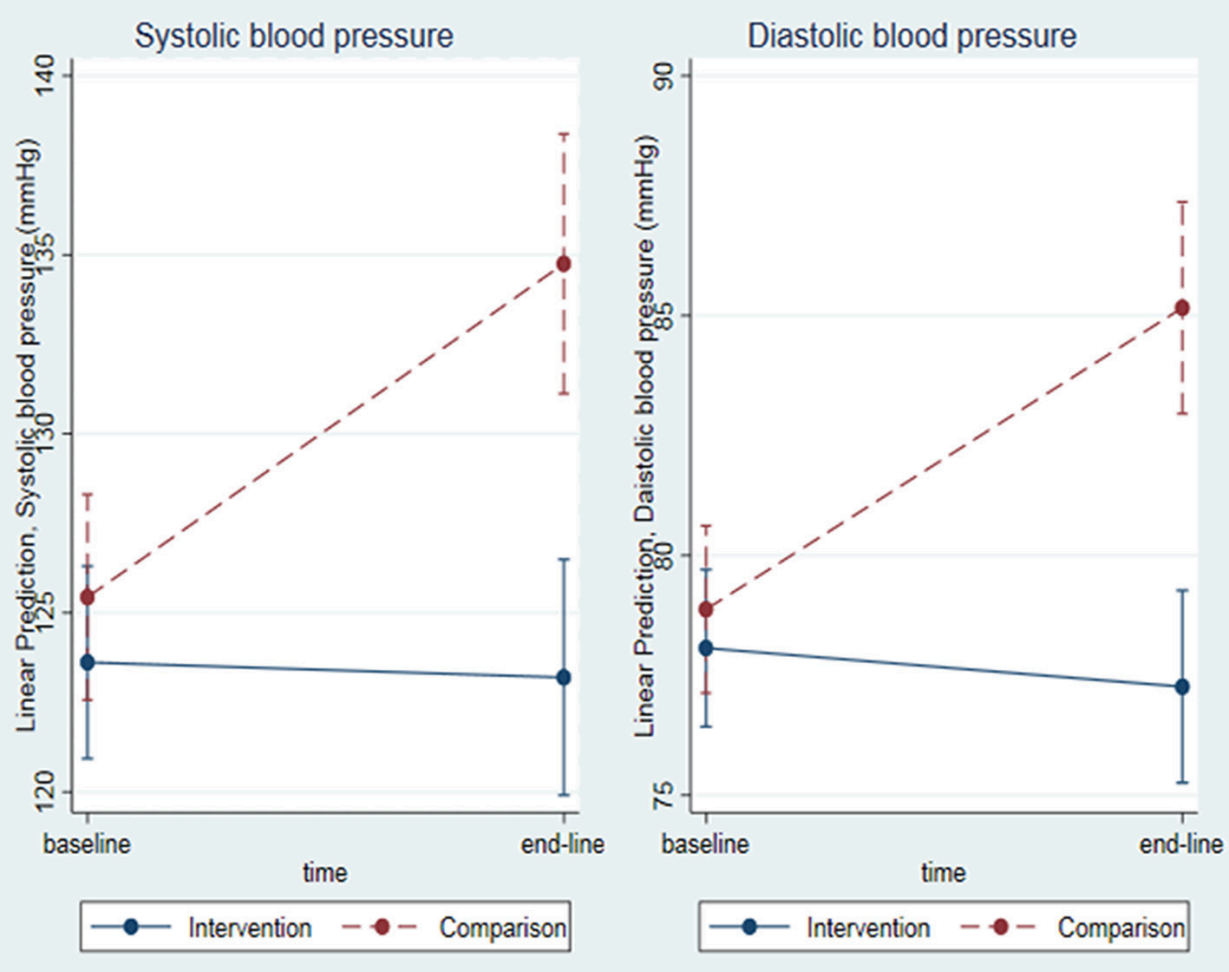
Mean end-line SBP and DBP among T2DM patients attending JUMC, Oct. 2017.

When adjusted for sociodemographic and clinical factors (including baseline SBP), mean end-line SBP was significantly lower in the intervention group, by 12 ± 3 mmHg (CI −17, −7; *P* = 0.000), than in the comparison group. Similarly, adjusted mean end-line DBP was significantly lower in the intervention group, by 8 ± 2 mmHg (CI −11, −5; *P* = 0.000; Figure 6).

### Anthropometric measurements

When adjusted for sociodemographic and clinical factors, mean differences between groups with respect to end-line BMI, waist circumference, and waist-to-hip ratio were not significant.

## Discussion

In this study, a DSME intervention was investigated as a critical component of diabetes management (10, 28). To accommodate low-literate patients and help them engage in self-management, the program was adapted to local context and used illustrative pictures and experience sharing. Our hypothesis is that DSME improves glycemic control over relatively longer time in rural African settings, where the lack of trained health-care providers and medical supplies is a major challenge (28).

Long-term glycemic control is one of the strongest clinical-outcome indicators of efficient diabetes management and health outcomes (7). The very limited research in Africa has provided inconsistent evidence regarding the clinical significance of DSME (7, 20, 21, 29, 47–49). The current study showed a significant mean HbA1c drop within both intervention and comparison groups. The drop in both groups is clinically meaningful since a drop of even <1% is associated with risk reduction for microvascular and macrovascular complications (50). However, the current study showed that the end-line mean HbA1c difference between groups was not significant. This non-significant difference between groups may be related to information spillover, low attendance rate, LTFU, variation in medication dose/type, and patients' short periods of contact with health providers. Similarly, other studies in Africa have not shown significant difference in HbA1c between intervention and comparison groups (21, 47). However, intervention provided over longer period resulted in significant differences in HbA1c between groups (20, 48, 49, 51). This indicates that DSME provided by well-trained nurses over longer periods of time may be effective for long-term glycemic control. We found significantly lower FBS at end-line in the intervention group, as adjusted for sociodemographic and clinical factors (including baseline values). This is similar to the finding of a study conducted in Egypt (47).

On the other hand, for blood pressure mean end-line SBP and DBP were significantly lower in the intervention group. These finding is consistent with a study conducted in South Africa (21). Even though a drop in blood pressure in diabetic patients is not directly related to glycemic control, it is associated with a clinically significant reduction in the risk of macrovascular and microvascular complications, endpoint diabetes complications, and premature death (52).

The challenges of this study included a low attendance rate and LTFU, because only about half of the participants were present in all six DSME sessions. Transportation challenges, the rainy summer season during which the intervention was conducted, and poor or no network accessibility might be contributing factors. Another possible reasons for the low attendance rate may be transfer of some patients to other facilities and frequent and unexpected medication stock out during the intervention period. Logistical issues during transition from the old hospital to the newly built hospital, created a long gap between the baseline and commencement of the intervention, which also may have contributed to LTFU.

This study has several limitations. First, recruiting both the intervention and the comparison group from the same hospital probably created some information spillover, either verbally or through sharing instructional books and fliers. Second, the gap between the baseline survey and commencement of the intervention could affect measurements. Third, even though we randomized participants based on residence in either Jimma city or districts, there might have been unforeseen selection biases. Fourth, because some of the data collectors were recruited from the same hospital, there might have been social desirability bias.

Despite such limitations, the study contributes important insights for diabetes self-management interventions in Africa. For Sub-Saharan African countries, this study contributes a DSME program supported by instructional handbooks and fliers with illustrative pictures and experience sharing among diabetic patients, are means to accommodate patients with low levels of literacy. The study indicates that it may be effective to use nurses as front-line diabetic-care providers and educators. In addition, the findings show significant improvement in HbA1c in both groups. However, we did not achieve our primary outcome of increasing by 15% the proportion of diabetes patients in the intervention group with higher glycemic control than patients in the comparison group. The intervention demonstrated significantly lower FBS and SBP and DBP, which are clinically meaningful.

A carefully developed and contextualized DSME program incorporating generous use of pictures and experience sharing is promising in the Ethiopian context. To further strengthen such efforts, additional strategies for delivery, including audio, video messages, teach back and combination of group-based and one-to-one approaches over a longer period that could accommodate low-literate diabetic patients could be investigated (7, 19, 28, 29).

To achieve even more control and reduce the risk of contamination, future studies of DSME should consider designs that incorporate a comparison group from a different hospital or community. In addition, to determine long-term glycemic control, introduction of HbA1c test laboratory facility to at least specialized, referral, and general hospitals in the country is suggested.

## Availability of data and materials

The raw data supporting the conclusions of this manuscript will be made available by the authors, without undue reservation, to any qualified researcher upon reasonable request.

## Ethics statement

All procedures performed in studies involving human participants were in accordance with the ethical standards of the institutional and/or national research committee and with the 1964 Helsinki declaration and its later amendments or comparable ethical standards. Before the commencement of the study, ethical approval was secured from the Norwegian Regional Committee for Medical and Health Research Ethics (REK) and the Jimma University Ethical Review Board. Written informed consent was obtained from all individual participants included in the study.

## Author contributions

All authors made a substantial contribution, from proposal development and study design to the writing of the manuscript. Specifically, FH conceived the idea and developed the study proposal, did the fieldwork, analyzed data, interpreted the findings, and led the manuscript writing. PH was involved in proposal development, planning the fieldwork, data analysis and interpretation, manuscript editing and manuscript review. AM was involved in proposal development, planning the fieldwork, data interpretation, manuscript editing and manuscript review. All authors approved this manuscript for publication.

### Conflict of interest statement

The authors declare that the research was conducted in the absence of any commercial or financial relationships that could be construed as a potential conflict of interest.

## Abbreviations

ADA: American Diabetes Association
BMI: body mass index
DSME: diabetes self-management education
FBS: fasting blood sugar
HbA1c: glycated hemoglobin
HFIAS: household food insecurity access scale
IDF: International Diabetes Federation
JUMC: Jimma University Medical Centre
LTFU: lost to follow-up
mg/dL: milligram per deciliter
mmHg: millimeter mercury
NORHED: The Norwegian Programme for Capacity Development in Higher Education and Research for Development
OHAs: oral hypoglycemic agents
RBS: random blood sugar
REK: Norwegian Regional Committee for Medical and Health Research Ethics
SACCADE: Strategic and Collaborative Capacity Development in Ethiopia and Africa
T2DM: type 2 diabetes mellitus
UKDPS: United Kingdom Diabetes Prospective Study
USD: United States Dollar.
